# Burden of Hospitalizations Related to Pneumococcal Infection in Spain (2016–2020)

**DOI:** 10.3390/antibiotics12010172

**Published:** 2023-01-14

**Authors:** Ruth Gil-Prieto, Nizar Allouch, Isabel Jimeno, Valentín Hernández-Barrera, Raquel Arguedas-Sanz, Ángel Gil-de-Miguel

**Affiliations:** 1 Preventive Medicine and Public Health, Rey Juan Carlos University, 28922 Madrid, Spain; 2 Primary Care Health Center Isla de Oza, Vaccine Responsible of SEMG, 28035 Madrid, Spain; 3 Department of Business Economics and Accounting, Universidad Nacional de Educación a Distancia UNED, 28004 Madrid, Spain; 4 CIBER of Respiratory Diseases (CIBERES), Instituto de Salud Carlos III, 28029 Madrid, Spain

**Keywords:** pneumococcal infection, epidemiology, hospitalizations, pneumococcal pneumonia

## Abstract

Pneumococcal infection strongly contributes to morbidity and mortality in Spain. A total of 253,899 hospitalizations related to pneumococcal infection occurred from 2016 to 2020. Fifty-eight percent were men, the mean age was 67 years old, and the average length of hospitalization was 12.72 days. The annual hospitalization rate was 10.84 hospitalizations per 10,000 population, increasing significantly with age, reaching 65.75 per 10,000 population in those aged >85 years. The hospitalization rates for pneumococcal pneumonia, sepsis, and meningitis were 2.91, 0.12, and 0.08 hospitalizations per 10,000, respectively, and reached the highest value in those aged >85 for pneumococcal pneumonia and sepsis, with 22.29 and 0.71 hospitalizations per 10,000, respectively, and in children up to 1 year old for pneumococcal meningitis, with 0.33 hospitalizations per 10,000. The total number of deaths during the study period was 35,716, with a case-fatality rate of 14.07%. For pneumococcal pneumonia, sepsis, and meningitis, the case-fatality rates were 8.47%, 23.71%, and 9.99%, respectively. The case-fatality rate increased with age and did not vary by sex. The annual cost of these hospitalizations was more than EUR 359 million. There is therefore a high burden of disease and mortality caused by pneumococcal infection in our country, especially in elderly individuals.

## 1. Introduction

*Streptococcus pneumoniae* (pneumococcus) is a leading cause of acute respiratory and invasive infections in all ages [1] and leads to a high number of hospitalizations and costs, especially in groups considered at risk [2]. This includes lifestyle factors (smoking, alcohol abuse, being underweight, having regular contact with children, and poor dental hygiene), comorbid conditions (chronic respiratory and cardiovascular diseases, cerebrovascular disease, Parkinson’s disease, epilepsy, dementia, dysphagia, HIV, or chronic renal or liver disease) and age. Immunosenescence [3] makes age the most important risk factor, dramatically increasing the morbimortality of pneumonia. Among the community-acquired pneumonias with an identified microbiological cause, *Streptococcus pneumoniae* and respiratory viruses are the most frequently detected pathogens [4].

Although the trend in recent years points towards a decrease in the incidence of pneumococcal pneumonia due to preventive measures such as vaccination, pneumococcal disease continues to be associated with high rates of morbidity and mortality and long periods of hospitalization, which translates into high health costs [5].

In a recent retrospective multicenter study on pneumococcal infection in Spanish hospitals between 2008 and 2017 [6], the mean patient age was 63 years, and pneumococcal pneumonia was the cause of 64% of pneumococcal disease, with a mortality of 7%. *S. pneumoniae* is also responsible for invasive pneumococcal diseases (IPDs), such as meningitis and sepsis, and more common noninvasive illnesses, such as sinusitis and otitis media.

Four vaccines have been commercialized for protection against pneumococcal disease in Spain: a 23-valent polysaccharide vaccine (PPV-23), three pneumococcal conjugated vaccines (PCV) (a 10-valent conjugated vaccine (PCV10) and a 13-valent conjugated vaccine (PCV13) and the recently approved 20-valent conjugated vaccine). The Spanish vaccination policy recommends the administration of PCV13 for children (two doses of primary vaccination and one booster), for high-risk adults (PCV13 followed by PPV23), for those older than 2 years who are at risk due to underlying conditions (a dose of PPV23 after having completed the usual immunization with PCV13), and for all adults aged 65 years and older (one dose of PPV23) [7,8].

Due to the expected indirect effects of PCV13 vaccination of the pediatric population on the reduction of the pneumococcal disease burden in adults, policy has moved away from routinely recommending PCV13 for elderly individuals. In the last months of 2022, after the approval of the 20-valent conjugated vaccine, some regions in Spain began shifting their vaccination policies and recommending vaccination in the elderly with PCV20.

The objective of this study was to provide population-based estimates of the burden of hospitalization for pneumococcal infection (PI) and specifically for pneumococcal pneumonia (PP), pneumococcal sepsis (PS), and pneumococcal meningitis (PM) in the general population in Spain during a five-year period (2016–2020).

## 2. Results

In total, 253,899 patients were hospitalized in Spain due to pneumococcal infection from 2016 to 2020. Of those, 58% (146,081 cases) were men and 42% (107,818) were women. The mean age was 67.00 (CI 95%: 66.92–67.08) years old, and the average length of stay was 12.72 days (CI 95%: 12.67–12.80).

**Figure 1:**
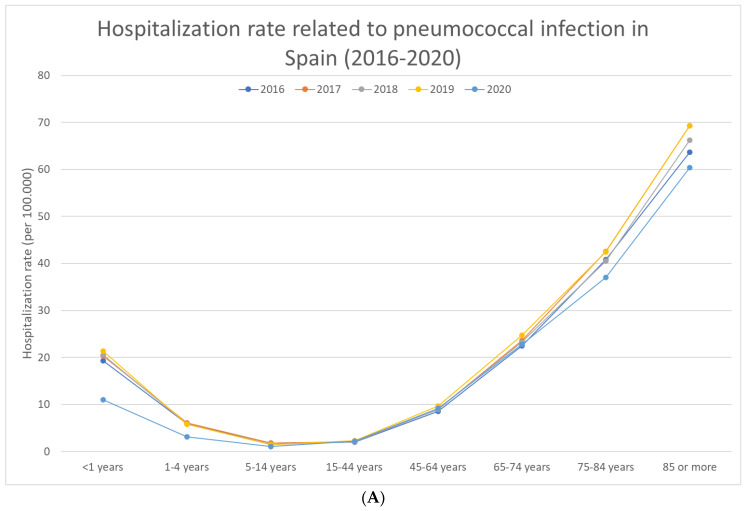

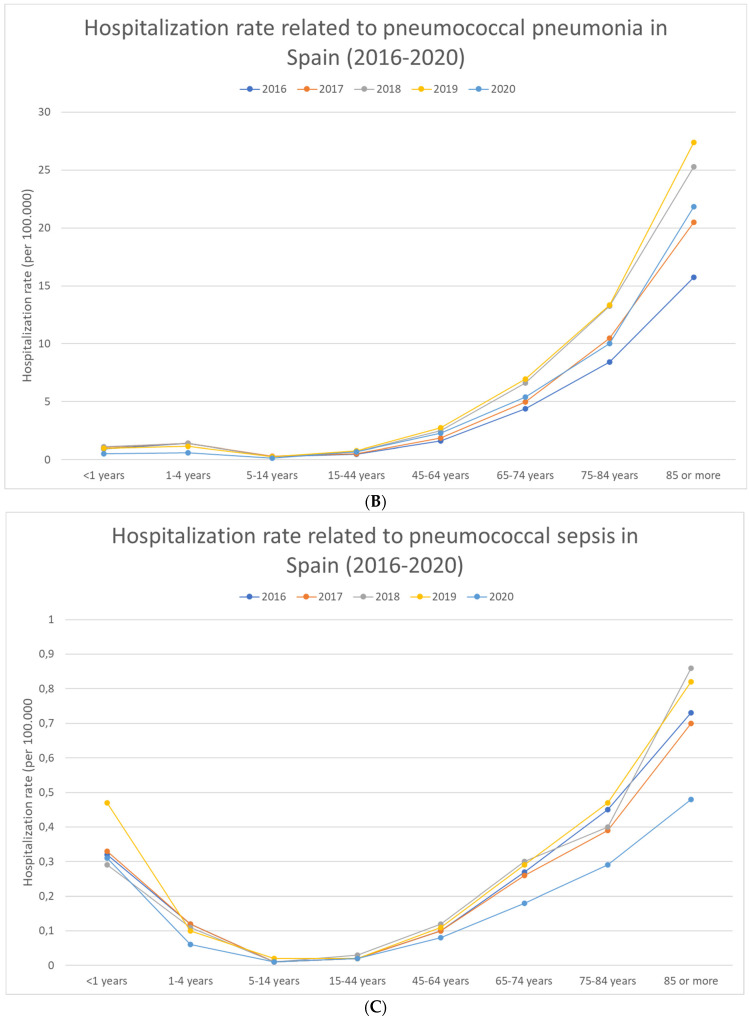

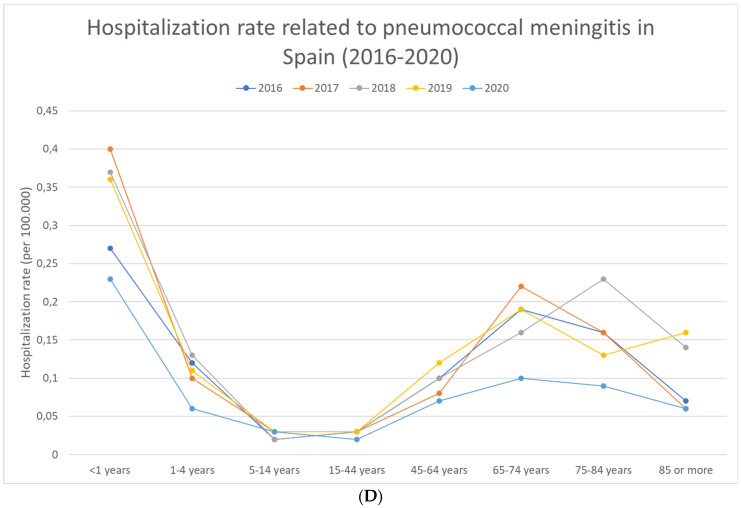
(**A**): Hospitalization rate related to pneumococcal infection by age group and year of study in Spain (2016–2020). (**B**) Hospitalization rate related to pneumococcal pneumonia by age group and year of study in Spain (2016–2020). (**C**) Hospitalization rate related to pneumococcal sepsis by age group and year of study in Spain (2016–2020). (**D**) Hospitalization rate related to pneumococcal meningitis by age group and year of study in Spain (2016–2020).

The overall annual hospitalization rate was 10.84 hospitalizations per 10,000 population (CI 95%: 10.8–10.88), with significant differences by age group (*p* < 0.001 in all diagnoses and in both females and males) (Table 1, Figure 1A). The hospitalization rate in children up to 1 year of age was 18.6 cases per 10,000 population (CI 95%: 17.99–19.21), decreasing with age until adolescence (1.5 cases per 100,000, CI 95%: 1.45–1.55 in the 5–14 year old group) and then increasing again with age, reaching 65.75 cases per 10,000 population (CI 95%: 65.17–66.33) in those aged 85 or over (Table 1).

Pneumococcal pneumonia accounted for 68,189 hospitalizations, pneumococcal meningitis accounted for 2763, and sepsis accounted for 1761, with 57%, 60%, and 53% of the hospitalizations occurring in males, respectively. The differences by year were statistically significant in most of the age groups (*p* < 0.001), mainly because of the decrease in the hospitalization rate in 2020 (Figure 1). The hospitalization rates for pneumococcal pneumonia, sepsis, and meningitis were 2.91 hospitalizations per 10,000 (CI 95%: 2.89–2.93), 0.12 hospitalizations per 10,000 (CI 95%: 0.12–0.12), and 0.08 hospitalizations per 10,000 (CI 95%: 0.08–0.08), respectively. The highest values were reached in those aged >85 for pneumococcal pneumonia, with 22.29 hospitalizations per 10,000 (CI 95%: 21.95–22.63), and sepsis, with 0.71 hospitalizations per 10,000 (CI 95%: 0.65–0.77), and in those up to 1 year old for pneumococcal meningitis, with 0.33 hospitalizations per 10,000 (CI 95%: 0.25–0.41).

The hospitalization rates were generally higher in males than in females (Table 1 and Table 2), reaching statistically significant differences in those aged <1 year and all groups aged older than 15 years for all hospitalizations and pneumococcal pneumonia. In sepsis, the differences were significant in those older than 15 years of age. In pneumococcal meningitis, statistical significance was only reached in the <1-year-old, 15–44-year-old, and 65–74-year-old groups.

The total number of deaths among patients hospitalized with pneumococcal infection during the 5-year study period was 35,716. Of those, 5,774 were related to pneumococcal pneumonia, 655 to sepsis, and 176 to pneumococcal meningitis. This corresponded to a case-fatality rate of 14.07% (CI 95%: 13.93–14.2) for pneumococcal infection-related hospitalizations (Table 1) and of 8.47% (CI 95%: 8,26–8,68), 23.71% (CI 95%: 22.15–25.32), and 9.99% (CI 95%: 8.66–11.46) for pneumococcal pneumonia, sepsis, and meningitis, respectively (Table 2).

The case-fatality rate increased with age (*p* < 0.001), reaching a higher value in the >85-year-old group, with 19.07% (CI95%: 18.72–19.42) for PI. For pneumococcal pneumonia, sepsis, and meningitis, the highest case fatality rate was also found in those aged >85, with 14.15% (CI 95%: 13.62–14.69), 34.97% (CI 95%: 31–39.11), and 47.3% (CI 95%: 36.21–58.59), respectively (Table 2).

There were no statistically significant differences in the case fatality rates by year during the study period, except for those patients aged 65 or over with a diagnosis of pneumococcal pneumonia; in this age group, the case-fatality rate was significantly higher in 2020.

The mean cost per hospitalization was EUR 7081 (CI 95%: 7044–7119). When gathering data for diagnosis, EUR 5247 (CI 95%: 5195–5299) was the mean cost per PP hospitalization, EUR 9169 (CI 95%: 8761–9577) was the mean cost for sepsis, and EUR 12,608 (CI 95%: 12,107–13,109) was the mean cost for meningitis. The estimated cost of these hospitalizations per year was more than EUR 359 million in total, with EUR 71, 5, and 4.4 million for pneumococcal pneumonia, sepsis, and meningitis, respectively.

## 3. Discussion

In this study, we provide data on the burden of hospitalizations for pneumococcal infection in Spain. Our results show that these diseases continue to pose a high burden of both morbidity and mortality and health care costs, with an annual general hospitalization rate of 10.84 hospitalizations per 10,000 population and reaching 65.75 per 10,000 population in those aged >85, with an annual cost of more than EUR 359 million.

The hospitalization rates obtained in this study are in line with previously published hospitalization rates (1.09/1000) reported for pneumococcal pneumonia in adults older than 50 years old in Spain for the period 2003–2007 [9] and with the decreasing trend in hospital incidence of pneumococcal pneumonia reported in the last decade [6].

When comparing our results with other European countries, Italy showed a general hospitalization rate of 753/100,000 inhabitants after the introduction of the pneumococcal conjugate vaccine in children [10], whereas Norway observed a decrease in the incidence of pneumococcal pneumonia from 50.8 cases per 100,000 in 2008 to 35.6 cases per 100,000 in 2009 in those aged 65 years and older [11]. A study performed in Portugal showed an average annual rate of hospital admissions for adults with community-acquired pneumonia of 3.61 per 1000 total population, rising to 13.4 for those aged ≥65 years and increasing between 2000 and 2009 [12]. This increase is believed to be related to the impact of age on the increase in admissions for community-acquired pneumonia (CAP), as the average age grew from 70.1 years in 2000 to 73.6 years in 2009. In general, a higher hospitalization rate is seen in Spain. This phenomenon was previously observed in a recent systematic review, which highlights Spain as having the highest prevalence of pneumococcal pneumonia among Southern European countries. [13,14]. The multiple changes in vaccinations in the last two decades and the consequent impact on the pneumococcal disease-related hospitalization rate have made it difficult to compare between countries due to the different time ranges of the studies.

The hospitalization rates for pneumococcal disease remained stable from 2016 to 2019 in our study, similar to what was found in northern France, where the incidence of hospitalizations related to invasive pneumococcal disease remained stable from 2014 to 2018, with no significant increase in pneumococcal serotypes not included in the vaccines [15,16]. The decrease in the hospitalization rates shown in 2020 may have been due to the COVID-19 pandemic. This effect was also observed among seven US children’s hospitals, in which the cumulative incidence of invasive pneumococcal disease decreased by 46% in 2020 vs. 2017–2019 [17]. Since SARS-CoV-2 is a respiratory virus, there are some concerns that COVID-19 can increase susceptibility to pneumonia and invasive pneumococcal diseases. However, early reports during the pandemic demonstrated reduced IPD rates [18], and coinfections of these two pathogens were infrequent [19]. It is thought that the reduction in pneumococcal disease in children observed during the COVID-19 pandemic was mainly due to the reduction in the incidence of these seasonal respiratory viruses rather than to reductions in transmission of pneumococcus [16].

Similar to previous studies, the high direct costs of pneumococcal disease were confirmed, with more than EUR 359 million annually. In 2011, a study on the risk of hospitalization due to pneumococcal disease in Spain [20] reflected a direct economic burden of PP of EUR 47 million, with EUR 57 million accounting for all invasive pneumococcal disease. This is consistent with the data offered by another study in 2015 on the impact of four vaccine-preventable diseases in older adults in Spain [5] and Italy, where, despite reductions in pneumococcal disease-related expenditures following the introduction of PCV13, there continues to be an important economic burden associated with pneumococcal disease [21]. In Norway, a recent study indicated that the burden of noninvasive pneumococcal pneumonia hospitalization had a substantial impact on the health and health care use of the 50+ population in Norway, despite the childhood immunization program [22].

It has been hypothesized that adult pneumococcal pneumonia caused by the serotypes included in the conjugate vaccine also declined following the introduction of pediatric PCVs in national immunization programs, possibly due to the indirect effects of pediatric PCVs. The introduction of PCVs into the US routine infant vaccination schedule led to important reductions in the burden of IPD and noninvasive pneumonia among vaccinated and unvaccinated populations [23], but despite the herd protection observed in US adults, noninvasive pneumococcal CAP and vaccine-type pneumococcal CAP remain a burden in older adults [24,25]. Data from Italy showed that, although the pneumococcal pediatric vaccination resulted in a decrease in hospitalizations in children, the expected indirect effect in the elderly was not reported, leading to the recommendation to extend the vaccination to subjects >64 years of age [10,26]. Similarly, in Israel, pediatric pneumonia hospitalization rates have continued to decline since the introduction of PCV without increasing the frequency of complications, but pneumococcal serotype distribution shifted in parallel, confirming the efficacy of PCV and supporting the evidence to include more serotypes in the next generation of PCV [27]. The proportion of adult pneumococcal pneumonia caused by PCV13 serotypes in Japan also declined after pediatric PCV introduction into national immunization programs, possibly due to the indirect effects of pediatric PCVs [28]. PCV13 implementation in France led to a major reduction in the incidence of invasive pneumococcal disease. However, a rebound in cases among children and adults since 2015, driven by several emerging non-PCV13 serotypes, jeopardizes the long-term PCV benefits [10,29]. In the context of a robust pediatric PCV13 immunization program, the PCV13 vaccination of adults aged 65 years or older was associated in the US with significant reductions in hospitalizations for all-cause pneumonia and low respiratory tract infections. Vaccinating older adults with PCVs may provide broader public health benefits against pneumonia hospitalizations [30]. These findings indicate the importance of having conjugate vaccines with high serotype coverage and might guide policymakers in the selection of future pneumococcal vaccines.

PCV13 has been widely used in the last decade and has been associated with an important decrease in the rate of pneumococcal pneumonia in comparison to PPV23. A study in older US veterans showed a 31% decrease in the rate of pneumococcal pneumonia with the conjugate vaccine in comparison to PPV23, recommending routine vaccination with pneumococcal conjugate vaccines in all older adults [31]. Sequential PCV13/PPV23 vaccination was more effective at preventing pneumococcal CAP among elderly individuals aged 65–74 years than single-dose PCV13 or single-dose PPV23 [32]. More recently, the immunogenicity of PCV20 in adults has been demonstrated in several clinical trials that showed that PCV20 administered as a single dose induced robust immune responses and was well tolerated [33].

The present study was not designed to evaluate the efficacy of preventive measures. The early direct effect of PCV13 and PPV23, since their inclusion in the regional calendars of immunocompetent adults, has been broadly discussed [34]. Long periods of time are needed to see the indirect effects of PCV13 in childhood; approximately 3 years are needed to see a reduction of 50% in the incidence of the cases caused by the serotypes included in this vaccine and a decade to be nearly eradicated [35]. According to data from De Miguel et al. [34], the three serotypes most frequently involved in IPD and causing high mortality in 2019 in Spain were 8 (18.72%), 3 (13.2%), and 22F (5.3%). Compared with PCV13 serotypes, the additional 2 and 7 serotypes covered by PCV15 and PCV20 would cover 550,475 and 991,220 annual pneumococcal disease cases, as well as 1425 and 3226 annual deaths, respectively, with savings ranging from USD 903 to USD 1928 million in the United States [36]. It is estimated that the new PCV15 and PCV20 vaccines would achieve coverage of 15% and 24% of all-cause pneumonia and 43% and 70% of pneumococcal pneumonia by studying the most prevalent serotypes in the 2016–2018 period, respectively [14]. The use of PCV20 among adults currently eligible for PPV23 in England would substantially reduce the burden of pneumococcal disease, with a modest budgetary impact [37]. The Advisory Committee on Immunization Practices (ACIP) recommends the use of a pneumococcal conjugate vaccine (either PCV20 or PCV15) for adults 65 years of age or older, those risk groups who have not previously received a pneumococcal conjugate vaccine, or those whose previous vaccination history is unknown. If PCV15 is used, the recommendation is that it should be followed by a dose of PPV23 [38]. Considering that a single dose of PCV20 would cover the majority of pneumococcal disease in Spain and improve vaccine compliance [39], the recommendation by the NeumoExperts group is using a single dose of PCV20. Some Spanish regions have recently recommended the use of a single dose of PCV20 [40,41].

Regarding the limitations of this study, it is important to note that, despite gathering all cases of hospitalizations related to pneumococcal disease, we were unable to take into account the cases that were managed in primary care and did not provide information on microbiological confirmation; thus, our results may have underestimated the true impact of pneumococcal pneumonia. Incidences were not given in this study, only hospitalization rates. There is a potential undercoding of clinical variables due to the limited number of codes for secondary diagnoses or to deficiencies in the preparation of hospital discharge reports. The MBDS encodes hospital admissions; thus, there could potentially be data redundancy for patients who had been hospitalized more than once during the period analyzed. This potential redundancy could have affected the point estimates of the prevalence (overestimation) and lethality (underestimation) of the disease. However, due to the large number of records in the database, it is unlikely that a certain percentage of redundant data could have altered the general trends in hospitalization and death rates in the long term. Despite this limitation, the MBDS is currently one of the most valuable tools available for clinical and epidemiological research. The main strength of our study is derived from the use of the MBDS database, which, due to its large sample size, provides high statistical power and representativeness when analyzing clinical variables such as case-fatality rate. Additionally, the MBDS is subject to a high-quality data audit at the state level. This makes it a valuable tool for epidemiological analyses of respiratory diseases such as pneumonia [42]. The transition from ICD-9-CM to ICD-10-CM coding, starting in 2016, probably had some impact on the epidemiological analysis, affecting the comparison with hospitalization and mortality data previously published by this research group. It may be necessary to wait for longer-term epidemiological data to assess the true dimension of this transition [43]. However, a strength of this study is that the data were gathered nationwide over 5 years. This will make it easier to calculate the real impact of future vaccination on hospitalizations due to pneumococcal disease in the Spanish population. On the other hand, the use of the national hospital discharge database means that the reliability of hospital surveillance depends on the quality of the discharge reports and the precision of ICD-10 to detect cases of pneumococcal infection through the corresponding codes [44].

Continuous epidemiological surveillance is needed to assess the long-term effects of current preventive measures and the implementation in the coming years of new vaccines with more serotypes, such as PCV15 and PCV20, which will increase the potential coverage for children and adults.

## 4. Materials and Methods

In this retrospective study, the Spanish National Hospital Data Information System (Minimum Basic Data Set; MBDS) of the Spanish Ministry of Health was used to collect all retrospective cases with a diagnosis of pneumococcal disease from 1 January 2016 to 31 December 2020. The MBDS provides a complete registry of hospitalizations, as it covers approximately 98% of public hospitals, offering health coverage to approximately 99.5% of the Spanish population and is validated for data quality and overall methodology by the Spanish Ministry of Health. It is considered a valuable system for the epidemiological analysis [45] of disease, according to the 10th Clinical Revision of the International Classification of Diseases (ICD-10-CM), in any diagnostic position were included. The ICD-10-CM codes used were J13—Pneumonia due to Streptococcus pneumoniae, J86.9—Pyothorax without fistula, J91.0—Malignant pleural effusion, J91.8—Pleural effusion in other conditions classified elsewhere, I30.1—Infective pericarditis, J98.11—Atelectasis, A 40.3—Sepsis due to Streptococcus pneumoniae, and G 00.1—Pneumococcal meningitis.

### Statistics

The annual hospitalization rates were calculated. For the rates, we used data on the population covered by hospitals included in the MBDS information system, adjusted for population figures obtained from municipal registers as the denominators. It was assumed that the distribution by age of the population covered by these public hospitals was equal to the general population. The case fatality rate, which reflects the severity of the cases, was calculated by dividing the number of deaths by the total number of hospitalizations (%). The average length of stay (ALOS) was calculated (total length of stay/total number of hospitalizations).

The chi-square test was used to assess significant differences in proportions. Poisson regression models were used to assess the differences in the rates of hospitalization during the study period by age group and sex. To assess the differences in the case fatality rates, logistic regression was used.

The cost of these hospitalizations to the health care system is estimated by the Ministry of Health. The cost was calculated by taking into account the diagnosis cost group, the total cost, and the number of discharges. The diagnostic cost group was based on the diagnosis-related groups (DRGs) for hospitalized patients based on the ICD classification, age, sex, and resource consumption. Each group has a similar weight in hospital costs and can be applied to each related patient. The DRG calculations were performed by 3 M with the Core Grouping System software [46].

For all tests, the significance level used was *p* < 0.05. Statistical analyses were performed with SPSS 27 and Stata 16.1 software.

The patient data were anonymized and deidentified prior to analysis. The local ethics committee (Rey Juan Carlos University Research Ethics Committee) ruled that no formal ethical approval was required for this study.

## Figures and Tables

**Table 1:** Hospitalization rate and case-fatality rate by sex and age group due to pneumococcal infection in Spain (2016–2020).

Sex	Age Group	Hospitalization Rate/per 10,000 (CI 95%)	*p*-Value	Case-Fatality Rate/% (CI 95%)	*p*
**Male**	**<1 years**	20.22 (19.33–21.11)	0.000	2.03 (1.47–2.72)	0.014
	**1–4 years**	5.29 (5.07–5.51)	0.000	1.43 (1–1.98)	0.736
	**5–14 years**	1.49 (1.42–1.56)	0.000	1.4 (0.94–2.01)	0.170
	**15–44 years**	2.47 (2.42–2.52)	0.000	4.52 (4.15–4.93)	0.804
	**45–64 years**	11.03 (10.92–11.14)	0.000	12.86 (12.53–13.21)	0.360
	**65–74 years**	31.51 (31.17–31.85)	0.453	15.87 (15.49–16.27)	0.007
	**75–84 years**	56.26 (55.68–56.84)	0.000	16.65 (16.26–17.04)	0.000
	**85 or over**	90.93 (89.75–92.11)	0.000	18.98 (18.48–19.49)	0.001
	**Total**	12.72 (12.65–12.79)	0.046	14.34 (14.17–14.52)	0.000
**Female**	**<1 years**	16.89 (16.05–17.73)	0.000	1.86 (1.28–2.63)	0.026
	**1–4 years**	5.42 (5.2–5.64)	0.000	1.16 (0.78–1.68)	0.722
	**5–14 years**	1.52 (1.45–1.59)	0.000	1.68 (1.16–2.36)	0.513
	**15–44 years**	1.74 (1.7–1.78)	0.000	6.54 (6–7.12)	0.165
	**45–64 years**	7.16 (7.07–7.25)	0.000	12.53 (12.12–12.95)	0.382
	**65–74 years**	16.15 (15.92–16.38)	0.000	13.06 (12.59–13.54)	0.135
	**75–84 years**	29.17 (28.81–29.53)	0.000	14.51 (14.08–14.95)	0.092
	**85 or over**	52.88 (52.24–53.52)	0.169	19.15 (18.67–19.63)	0.105
	**Total**	9.04 (8.99–9.09)	0.000	13.69 (13.49–13.9)	0.009
**BOTH**	**<1 years**	18.6 (17.99–19.21)	0.000	1.96 (1.54–2.45)	0.001
	**1–4 years**	5.35 (5.19–5.51)	0.000	1.3 (1–1.66)	0.994
	**5–14 years**	1.5 (1.45–1.55)	0.000	1.54 (1.17–1.97)	0.164
	**15–44 years**	2.11 (2.08–2.14)	0.000	5.35 (5.03–5.68)	0.264
	**45–64 years**	9.08 (9.01–9.15)	0.000	12.73 (12.47–13)	0.880
	**65–74 years**	23.37 (23.17–23.57)	0.022	14.84 (14.54–15.15)	0.004
	**75–84 years**	40.68 (40.36–41)	0.000	15.77 (15.48–16.06)	0.001
	**85 or over**	65.75 (65.17–66.33)	0.001	19.07 (18.72–19.42)	0.000
	**Total**	10.84 (10.8–10.88)	0.000	14.07 (13.93–14.2)	0.000

The *p*-value represents significant differences during the period 2016–2020.

**Table 2:** Hospitalization rate and case-fatality rate by sex and age group due to pneumococcal pneumonia, sepsis, and meningitis in Spain (2016–2020).

		Pneumococcal Pneumonia				Pneumococcal Sepsis				Pneumococcal Meningitis			
Sex	Age Group	Hospitalization Rate/per 10,000 (CI 95%)	*p*-Value	Case-Fatality Rate/% (CI 95%)	*p*-Value	Hospitalization Rate/per 10,000 (CI 95%)	*p*-Value	Case-Fatality Rate/% (CI 95%)	*p*-Value	Hospitalization Rate/per 10,000 (CI 95%)	*p*-Value	Case-Fatality Rate/% (CI 95%)	*p*-Value
**Male**	**<1 years**	1.06 (0.86–1.26)	0.248	0.97 (0.1–4.45)	0.532	0.38 (0.26–0.5)	0.352	0 (0–0)	-	0.42 (0.29–0.55)	0.200	2.44 (0.26–10.84)	-
	**1–4 years**	1.21 (1.11–1.31)	0.000	0.19 (0.02–0.88)	0.592	0.11 (0.08–0.14)	0.083	6.25 (1.79–15.75)	0.142	0.11 (0.08–0.14)	0.003	2.04 (0.22–9.14)	-
	**5–14 years**	0.25 (0.22–0.28)	0.037	1.3 (0.44–3.07)	0.181	0.01 (0–0.02)	0.066	0 (0–0)	-	0.03 (0.02–0.04)	0.628	0 (0–0)	-
	**15–44 years**	0.7 (0.68–0.72)	0.000	1.97 (1.53–2.51)	0.964	0.03 (0.02–0.04)	0.319	9.32 (5.05–15.55)	0.705	0.03 (0.02–0.04)	0.035	4.64 (2.1–8.89)	0.818
	**45–64 years**	2.71 (2.65–2.77)	0.000	6.5 (6.01–7.03)	0.034	0.14 (0.13–0.15)	0.445	20.84 (17.29–24.77)	0.181	0.09 (0.08–0.1)	0.088	6.98 (4.56–10.2)	0.344
	**65–74 years**	7.76 (7.59–7.93)	0.000	8.34 (7.76–8.95)	0.093	0.35 (0.31–0.39)	0.024	24.19 (20.05–28.73)	0.917	0.19 (0.16–0.22)	0.002	15.46 (11.03–20.85)	0.498
	**75–84 years**	15.65 (15.34–15.96)	0.000	10.11 (9.53–10.71)	0.004	0.6 (0.54–0.66)	0.269	26.05 (21.83–30.64)	0.322	0.17 (0.14–0.2)	0.076	19.63 (12.97–27.9)	0.514
	**85 or over**	31.02 (30.33–31.71)	0.000	14.17 (13.41–14.96)	0	0.98 (0.86–1.1)	0.025	30.49 (24.99–36.44)	0.39	0.08 (0.04–0.12)	0.938	50 (29.34–70.66)	0.111
	**Total**	3.41 (3.38–3.44)	0.000	8.84 (8.56–9.12)	0	0.15 (0.14–0.16)	0.012	22.29 (20.34–24.33)	0.84	0.08 (0.07–0.09)	0.000	10.18 (8.36–12.26)	0.906
**Female**	**<1 years**	0.78 (0.6–0.96)	0.176	1.39 (0.15–6.31)	0.532	0.3 (0.19–0.41)	0.710	10.71 (3.11–25.91)	0.275	0.23 (0.13–0.33)	0.240	4.76 (0.52–20.18)	-
	**1–4 years**	1.2 (1.09–1.31)	0.000	0.4 (0.08–1.29)	0.68	0.09 (0.06–0.12)	0.319	10.81 (3.76–23.69)	0.977	0.09 (0.06–0.12)	0.336	2.56 (0.28–11.36)	-
	**5–14 years**	0.25 (0.22–0.28)	0.000	0.34 (0.04–1.57)	0.371	0.01 (0–0.02)	0.833	0 (0–0)	-	0.02 (0.01–0.03)	0.280	3.7 (0.4–16.04)	-
	**15–44 years**	0.54 (0.52–0.56)	0.000	1.71 (1.24–2.3)	0.942	0.02 (0.02–0.02)	0.754	10.45 (4.8–19.42)	0.494	0.02 (0.02–0.02)	0.758	4.71 (1.61–10.8)	0.927
	**45–64 years**	1.73 (1.69–1.77)	0.000	4.2 (3.7–4.73)	0.23	0.07 (0.06–0.08)	0.193	17.47 (12.97–22.78)	0.047	0.09 (0.08–0.1)	0.692	5.9 (3.66–8.97)	0.468
	**65–74 years**	3.83 (3.72–3.94)	0.000	5.23 (4.61–5.9)	0.016	0.18 (0.16–0.2)	0.878	26.27 (20.75–32.41)	0.64	0.15 (0.13–0.17)	0.158	7.07 (4.02–11.45)	0.746
	**75–84 years**	7.73 (7.54–7.92)	0.000	8.29 (7.65–8.97)	0.001	0.26 (0.23–0.29)	0.033	27.93 (22.34–34.09)	0.515	0.14 (0.12–0.16)	0.168	15.45 (9.89–22.6)	0.815
	**85 or over**	17.83 (17.46–18.2)	0.000	14.13 (13.41–14.87)	0	0.58 (0.51–0.65)	0.648	38.87 (33.33–44.64)	0.633	0.11 (0.08–0.14)	0.337	46.3 (33.48–59.5)	0.644
	**Total**	2.44 (2.41–2.47)	0.079	7.97 (7.66–8.29)	0	0.09 (0.08–0.1)	0.100	25.87 (23.34–28.52)	0.25	0.07 (0.07–0.07)	0.621	9.79 (7.91–11.94)	0.936
**BOTH**	**<1 years**	0.92 (0.78–1.06)	0.000	1.14 (0.24–3.62)	0.435	0.34 (0.26–0.42)	0.646	4.62 (1.32–11.81)	34	0.33 (0.25–0.41)	0.719	3.23 (0.68–9.95)	0.695
	**1–4 years**	1.21 (1.14–1.28)	0.000	0.29 (0.08–0.78)	0.993	0.1 (0.08–0.12)	0.060	8.24 (3.76–15.49)	0.317	0.1 (0.08–0.12)	0.105	2.27 (0.48–7.09)	0.452
	**5–14 years**	0.25 (0.23–0.27)	0.000	0.83 (0.32–1.81)	0.098	0.01 (0.01–0.01)	0.113	0 (0–0)	-	0.02 (0.01–0.03)	0.277	1.67 (0.18–7.53)	-
	**15–44 years**	0.62 (0.6–0.64)	0.000	1.86 (1.53–2.24)	0.916	0.02 (0.02–0.02)	0.546	9.73 (6.08–14.62)	0.906	0.03 (0.03–0.03)	0.060	4.66 (2.5–7.92)	0.81
	**45–64 years**	2.22 (2.18–2.26)	0.000	5.6 (5.24–5.97)	0.022	0.1 (0.09–0.11)	0.167	19.71 (16.85–22.82)	0.028	0.09 (0.08–0.1)	0.135	6.45 (4.72–8.59)	0.227
	**65–74 years**	5.68 (5.58–5.78)	0.000	7.23 (6.79–7.68)	0.015	0.26 (0.24–0.28)	0.060	24.96 (21.59–28.57)	0.734	0.17 (0.15–0.19)	0.001	11.51 (8.63–14.95)	0.825
	**75–84 years**	11.09 (10.92–11.26)	0.000	9.38 (8.95–9.83)	0	0.4 (0.37–0.43)	0.033	26.74 (23.33–30.39)	0.693	0.15 (0.13–0.17)	0.027	17.39 (12.92–22.68)	0.768
	**85 or over**	22.29 (21.95–22.63)	0.000	14.15 (13.62–14.69)	0	0.71 (0.65–0.77)	0.069	34.97 (31–39.11)	0.299	0.1 (0.08–0.12)	0.393	47.3 (36.21–58.59)	0.191
	**Total**	2.91 (2.89–2.93)	0.000	8.47 (8.26–8.68)	0	0.12 (0.12–0.12)	0.003	23.71 (22.15–25.32)	0.596	0.08 (0.08–0.08)	0.000	9.99 (8.66–11.46)	0.991

The *p*-value represents significant differences during the period 2016–2020.

## Data Availability

All of the data generated or analyzed during this study are included in this published article and are available on reasonable request at https://pestadistico.inteligenc iadegestion.mscbs.es/publicoSNS/S/rae-cmbd. (accessed on 10 October 2021).
